# Impact of Food Allergies on the Food Safety and Life Quality of Adults in Spain

**DOI:** 10.3390/foods14060939

**Published:** 2025-03-10

**Authors:** Eulalia Antich Ferrer, Sandra Fernández-Pastor, Ana Guerrero

**Affiliations:** 1 Facultad de Ciencias de las Salud, Universidad Cardenal Herrera-CEU, CEU Universities, Alfara del Patriarca, 46115 Valencia, Spain; eulalia.antich@alumnos.uchceu.es; 2 Departamento Producción y Sanidad Animal, Salud Pública Veterinaria y Ciencia y Tecnología de los Alimentos, Universidad Cardenal Herrera-CEU, CEU Universities, Alfara del Patriarca, 46115 Valencia, Spain; sandra.fernandez@uchceu.es

**Keywords:** allergens, collective catering, consumers, FAQLQ, food allergy, food safety, food services, labeling regulation, questionnaire, restaurants

## Abstract

Food allergies are increasingly frequent immune system reactions triggered by allergens present in food, which can affect quality of life. To investigate the impact of food allergies among Spanish adults and the influence of gender and age of diagnosis, an online survey using the shortened version of the Adult Food Allergy Quality of Life Questionnaire (FAQLQ) was conducted. A total of 134 participants with food allergies were enrolled in the exploratory study. Significant differences (*p* ≤ 0.050) were found in the perception of the emotional and social impact of food allergies on quality of life. Age of diagnosis (childhood, adolescence, or adulthood) had a greater influence on more variables than gender. Men reported greater fear of accidentally consuming something that could trigger an allergic reaction compared to women (*p* = 0.003), while women felt more excluded due to their allergies (*p* = 0.030). Overall, the perception of eating out was characterized by insecurity. The quality of life of individuals with food allergies could be improved through the use of pictograms on labels, menus, and increased training in the foodservice industry regarding allergens. Multiple additional investigations are recommended to generalize current findings.

## 1. Introduction

Adverse reactions to food are those clinically abnormal responses that are triggered after the ingestion, contact, or inhalation of food, its derivatives, or additives. These reactions can be classified as food intolerances or allergies [1].

Food intolerances are dose-dependent adverse reactions; in this case, symptoms occur only if a certain amount is consumed, and there is no involvement of the immune system [1,2].

On the contrary, food allergies are non-dose-dependent adverse reactions with a verifiable immune response in susceptible individuals. This immune response is triggered by contact with small molecules, mostly proteins or water-soluble glycoproteins, named allergens, with the immune system [1]. Food allergies can be classified according to their physiopathology into IgE-mediated, non-mediated, and mixed as well as both IgE and non-IgE-mediated. Symptomatology differs depending on the type of reaction. In IgE-mediated food allergies, there is prior sensibilization to the allergen, so that when exposed to it again, symptoms of an acute illness (Anaphylactic shock) occur [3]. The reactions are rapid in onset and the immune response is triggered in less than two hours after contact with the allergen [2]. Non-IgE-mediated food allergies, as opposed to IgE-mediated food allergies that can lead to multiorgan anaphylaxis, are a group of disorders of subacute or chronic presentation characterized by inflammatory processes affecting mainly the gastrointestinal tract [4]. These reactions can occur between 2 and 48 h after contact with the food [1,2]. Finally, mixed food allergies include eosinophilic gastrointestinal disorders such as eosinophilic esophagitis and dermatological conditions such as atopic dermatitis [4,5].

The exact number of people with food allergies is unknown. This may be due to the existence of multiple kinds of food sensitivities with variable symptoms and severities. In addition, the lack of uniformity in diagnostic methods may also contribute to this. The prevalence of food allergies varies widely in different geographical locations and different incidences are observed depending on the dietary habits of each region [6]. There might not be an exact number; however, different revisions made in the area estimate that the prevalence in Spain is between 1 and 3% in adults and between 4 and 6% in children [7]. Other sources estimate a prevalence up to 7.4%; it is difficult to determine an exact number due to the difficulty in diagnosis, the variety of symptoms [6], and the variations that occur with age that can modify the kind of food that triggers allergy [8]. For example, it can be observed that a higher incidence occurs at the age of two, which later goes down. Foods that more frequently cause allergies in children younger than five are eggs, milk, and fish; however, in adults, the food causing allergies is nuts, vegetables, and celery [6,9]. The most frequent food allergies in Spain match with those that are the most frequent in the rest of Europe [6]. Sicherer [10] points out that globally, the most affected population (reaching up to 10%) is industrialized westernized regions and children.

Once the allergens causing the allergic process are identified, the main treatment is avoiding the foods that may contain them from their diet. Different approaches are being explored as potential treatments, such as immunotherapy or monoclonal antibodies [9].

The operator in food business is the main one responsible for food safety [11] and acts to ensure that businesses must follow the general and specific laws that apply to them. In matters of substances that can cause food allergies and intolerances, the law requires a declaration as part of the mandatory information to be provided to the consumers [12,13].

Even though legislation differs from country to country, there are eight common allergens that are declared, following the specifications of the norm CODEX STAN 1-1985 and general norm for the labeling of prepackaged food of the *Codex Alimentarius* [14]. These allergens are milk, eggs, fish, crustaceans, wheat, soy, peanuts, and nuts. To these ones, others can be added according to the relevance in each country [6].

**Figure 1:**
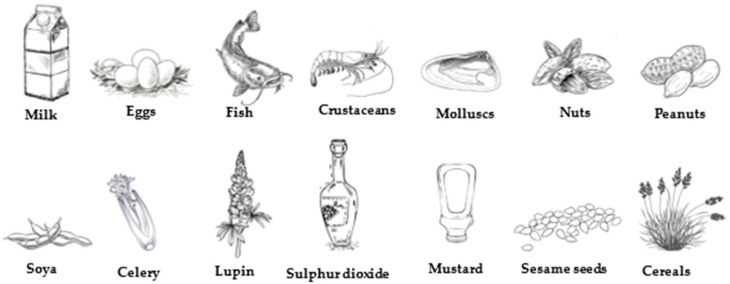
Allergens compiled in Regulation 1169/2011, [12].

At the European level, the Annex II of Regulation 1169/2011 on the provision of food information to consumers [12] lists the 14 substances that must be compulsorily declared on food labels (Figure 1). This regulation also establishes the content and presentation of other mandatory mentions and also requires that all of them be presented in a prominent place, in such a way as to be easily visible, clearly legible, and, if necessary, indelible. Thus, when it comes to the labeling of certain substances or products that cause food allergies or intolerances, it specifies that they must be included in the ingredients list and must be highlighted using a typographic composition that clearly differentiates them from the rest of the ingredients. In the absence of an ingredient list, the word “contains” must be included followed by the name of the substance.

This regulation not only applies to the packaging of foods but also to all food intended for final consumers, including those delivered by collective caterings and those intended for supply to collective caterers, as there are indications that most food allergy incidents originate from unpackaged foods [15]. In response to the mentioned regulation, Spain, in 2015, approved a General Norm related to the food information provided in unpackaged food for sale to the final consumers and mass caterers, for food packaged in places of sale at the request of the consumer, and for food packaged by retail trade holders [15].

However, despite the existence of a regulatory normative, the information provided to consumers is not presented in a homogeneous way in terms of allergens [16]. This can have a negative impact not only on people’s physical health but also on their socioemotional health, consequently affecting their quality of life [17]. In this sense, everyday situations such as shopping or cooking may be presented as real challenges, as they imply the need to continuously review labels that are sometimes non-uniform [16]. Participating in everyday activities involving food service, from birthdays to work events or school, also involves constant vigilance from allergy sufferers and their families. In these situations, many feel limited or socially excluded, which generates emotional effects on their mental health, such as depression, anxiety, bullying, or post-traumatic stress disorder [17,18].

The Food Allergy Quality Life Questionnaire (FAQLQ) is the most utilized tool for evaluating the quality of life related to the health of people with food allergies. This questionnaire is recommended by the European Academy of Allergy and Clinical Immunology as the reference standard evaluation [19] and is available in different languages. However, the length of the questionnaire can be a disadvantage associated with participation or the quality and reliability of the data. On occasion, participants do not answer either from boredom or disconnection; to solve Coelho [20], a shorter version for adults (FAQLQ-12) to minimize the negative aspects is proposed.

This questionnaire comes as an alternative for studying food allergies in adults. There are other FAQLQ questionnaires specifically indicated for children, adolescents, and parents [21,22,23]. All of them help participants, investigators, and doctors to obtain reliable and high-quality answers by utilizing a shorter questionnaire in less time.

## 2. Materials and Methods

This exploratory study was approved by the Ethics Committee for Biomedical Research of the Vice-Rectorate for Research of the UCH-CEU University (registration number: CEEI 23/498). An online and self-administered questionnaire was used. Comprehensive information about the study’s objectives and procedures was provided to all participants who provided written online informed consent. Survey answers were collected in the year 2024 from 15 February to 31 March.

To reach the specific study population, a convenience sampling approach was applied (non-probabilistic), in which the sample is chosen according to the accessibility criteria (adults who have a food allergy). It was combined with a snowball design. This type of sampling is suitable for exploratory studies (such as this one), although it limits the generalizability of its results due to the specificity of the sample, and the reliance on self-reported data may introduce bias, also making the sample non-generalizable as a limitation.

The Spanish Association of People with Food and Latex Allergies (Asociación Española de Personas con Alergia a Alimentos y Látex -AEPNAA) collaborates in this research to data collection. The association distributed the link that gave access to the survey among its members (which allowed access to a very specific and concrete population that in principle would meet the inclusion criteria). Also, AEPNAA published the link to the questionnaire on their social media platforms, promoting its dissemination among other people interested in food allergies, who are not members of the association. The snowball design allows diffusion by indication of another person that meets the initial sampling requirements [24,25].

The questionnaire consists of 36 questions, which were grouped in four blocks.

Firstly, the consent form for participation was distributed to all participants and signed. The consent was included in the first block of questions. This block also included filter questions to verify the inclusion criteria met (agreeing to participate in the survey, being over 18 years of age, and having a food allergy).

The second block consisted of 6 questions to characterize the sample socio-demographically (age, gender, place of residence, age of allergy diagnosis) and main allergies, as well as three dichotomous questions (yes/no) related to dietary and nutritional aspects (whether the sample has a healthy and balanced diet, nutritional deficiencies, or whether it is easy to replace foods that cannot be consumed).

The third block included the summarized and validated version of the FAQLQ questionnaire (Food Allergy Quality of Life Questionnaire) by Coelho [20], the Spanish version from the EuroPrevall project [26]. Table 1 details the selected questions. The scale used for answers to quantify how much the various actions collected in the questionnaire troubles, worries, or scares them had 7 different levels as possible answers, from not to extremely. (detailed in Table 2).

The last block included 11 questions (Table 3) related to food safety, food labeling, and the training of catering professionals. The cited intensity scale (for labeling section and safety perception eating out) and dichotomous (yes/no) and polytomous questions (yes/no/sometimes) were used for the other variables.

The survey results were collected using Microsoft Forms. All questionnaires (166) and answers were individually reviewed. The field data were entered on an Excel spreadsheet. After applying the inclusion criteria (accepting participation in the questions, being 18 years old or more, and having food allergies) and exclusion criteria (not accepting the consent to participate in the study, being younger than 18 years old, or not having food allergies) and checking for missing data and outliers, the sample was reduced to a final group of 134 surveys.

The SPSS for Windows Statistical package v.29.0.2.0 (IBM SPSS Statistics, SPSS Inc., Chicago, IL, USA) was used for the statistical analyses.

The first stage was running a descriptive analysis of the variables in order to obtain frequencies and percentages. It was considered two fixed factors (age of allergy diagnosis and gender).

Afterward, non-parametric tests (chi-square test for qualitative variables and Kruskal–Wallis for quantitative variables) were used to determine any statistically significant differences among the independent groups that compose each variable (sub-item question) for each fixed factor. This was followed by a statistical comparison using crosstabs procedures, including the Z test to check significant differences between the data of the variables, categories whose column proportions do not differ significantly from each other at the 0.05 level. Additionally, FAQLQ qualitative answers were transformed to quantitative using a seven-point Likert scale (1 = not; 2 = barely: 3 = slightly; 4 = moderately; 5 = quite; 6 = very; 7 = extremely); medians, standard deviation, and standard error of mean were calculated. Also, normality was explored using Kolmogorov–Smirnov and non-parametric tests were applied (U-Mann–Whitney and One way ANOVA of Kruskal–Wallis).

## 3. Results

### 3.1. Participant Description (Socio-Demographic Profile and Main Allergies)

The main socio-demographic and classificatory variables that define the participant population are shown in Table 4. Among the participants, there is a predominance of women, and the segments under 54 years old predominate. The age at which the participants were diagnosed with food allergies was mostly during childhood, and a similar percentage in adolescence and adulthood was reported.

**Figure 2:**
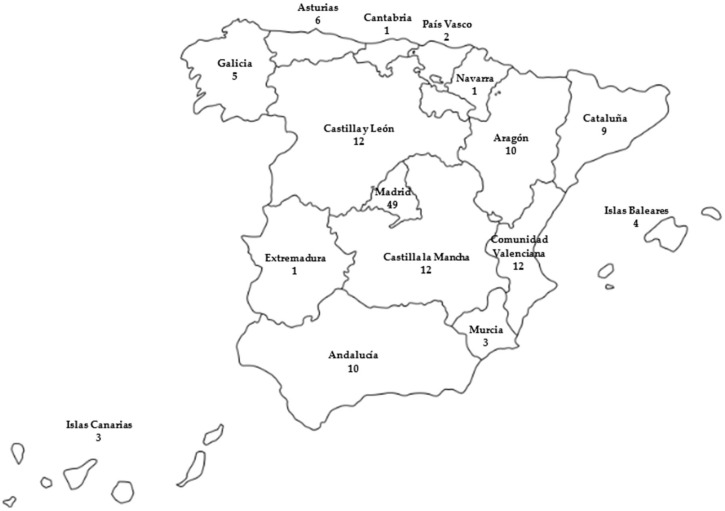
Distribution of the answers received in Spain (*n* = 134).

Figure 2 details the origin of the answers received. Predominantly, the participation came from certain regions, mostly in Madrid (*n* = 49), followed by Comunidad Valenciana (*n* = 12), Castilla la Mancha (*n* = 12), and Castilla y León (*n* = 12).

**Figure 3:**
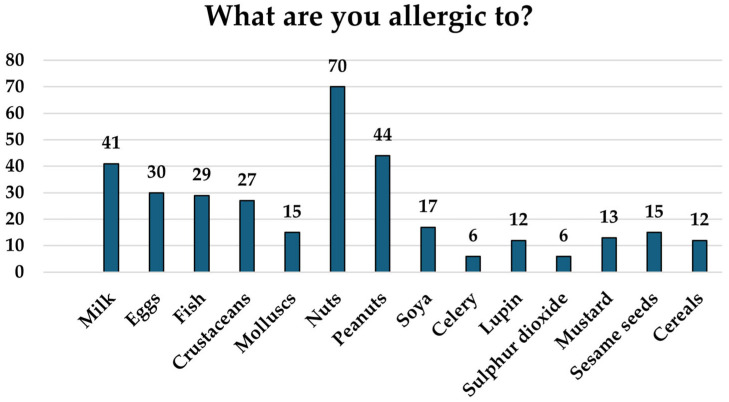
Food to which participants have allergies, shown as the number of participants that have an allergy to each food (*n* = 134).

Figure 3 shows the main foods that cause allergies. In first place are nuts and peanuts, which represent 52.2% and 32.8% of the total of respondents, respectively. They are followed by milk (30.6%) and eggs (22.4%).

### 3.2. Effect of Gender and Age of Diagnosis

According to the *p* values shown in Table 5, age of diagnosis influences more variables than gender does. Significant differences can be seen between genders related to age of allergy diagnosis (*p* < 0.001), as well as in variables related to emotional impact, such as “frightened of eating the wrong food” (FAQLQ 25) (*p* = 0.003) or “feeling excluded due to their allergy” (*p* = 0.030). Moreover, there is a tendency to significant differences between genders about the perception of nutritional deficiencies (*p* = 0.091) or FAQLQ 26 “being worried of having an allergic reaction when eating out of home” (*p* = 0.082) and use of food label pictograms (*p* = 0.095).

Regarding the age of diagnosis, there were significant differences between genders (*p* < 0.001). There were also significant differences in variables related to the emotional impact (FAQLQ 24) “the fear of having an allergic reaction” (*p* = 0.048) or (FAQLQ 25) “eating something that can cause a reaction” (*p* = 0.005). There were significant differences related to catering, which included changing places (*p* = 0.007). In addition, tendencies to significant differences were observed between age of diagnosis in variables related to the risk (FAQLQ 18) “underestimating food allergies” (*p* = 0.027).

### 3.3. Shorter Version of FAQLQ Answers

Table 6 compiles the general answers of participants to FAQLQ, presented as a percentage that quantifies the seven possible intensities of how much diverse actions collected in the questionnaire it troubles, worries, or scares. Most answers show high scores of intensities, which means that all of the variables studied affected in a “quite, very or extremely” way.

Table 7 and Table 8 detail the results of the variables whose answers showed a significant difference related to the fixed factors analyzed (gender and age of diagnosis).

Related to gender, significant differences were found in the answers to the question (FAQLQ 25 “frightened accidentally eating the wrong food”. Table 6 shows how men are more afraid to eat something by mistake than women were (*p* = 0.003).

Table 8, which is related to age of diagnosis, compiles significant differences in their answers, specifically in questions FAQLQ 24 “frightened of an allergic reaction” and FAQLQ 25 “frightened accidentally eating the wrong food”.

Participants diagnosed during adolescence were more afraid of having allergic reactions (FAQLQ 24) than those diagnosed as adults (*p* = 0.048). When eating something by accident (FAQLQ 25), those diagnosed as adults answered more frequently than those diagnosed during childhood, with the options “moderately or quite”. However, the ones who were afraid or more afraid of eating something by accident were those diagnosed during adolescence and childhood, respectively (*p* = 0.005).

According to the questions, FAQLQ 18 “Underestimate your allergy” tendency was observed (*p* = 0.052), and the group diagnosed during adolescence was more afraid of having allergic reactions.

### 3.4. Nutrition-Labeling and Food Safety When Eating out

In the nutrition section of the questionnaire, answers show that 79.9% of the participants think that it is possible to have a healthy and balanced diet even with their allergies. Overall, 20.9% of participants considered having some kind of nutritional deficiency, with women being the ones who answered “yes” the most. Nutritional deficiencies that came out more frequently were vitamins, such as A, D, and group B vitamins, minerals, as calcium, iron, phosphorus, zinc, and iodine, as well as fiber, omega 3 fatty acids, and docosahexaenoic acid (DHA). Overall, 56.0% of participants considered that the foods that caused allergies and could not be consumed were not easily replaceable for others.

Answers related to labeling are compiled in Table 9. Participants felt more upset when the labels showed the sentence “it may contain traces of …”; on the other hand, they found pictograms (for example, gluten free or dairy free) to be very useful. Regarding feeling safe eating out if they did not prepare their own food, the answers reflected a great feeling of insecurity.

Table 10 presents the results of the questions detailed above, converted into a seven-point Likert scale. It can be observed that the variables related to emotional impact (FAQLQ24, 25 and 26) and risk (FAQLQ 13, 14 and 18) are the sections with the highest values.

There were several places where participants felt that their allergies were underestimated; 95.52% pointed out restaurants and coffee shops (128/134), 41.79% educative centers (school/high school/university) (56/134), 35.82% hospitals (48/134), 35.07% museums (47/134), and 29.10% amusement parks or workplaces (39/134).

**Figure 4:**
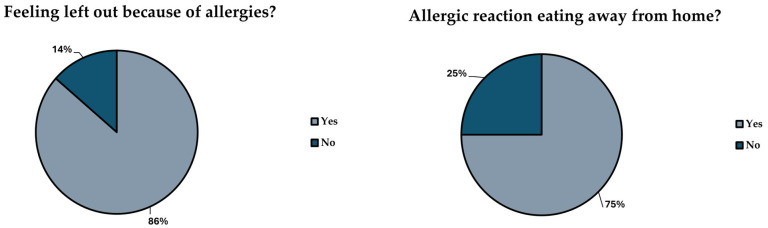

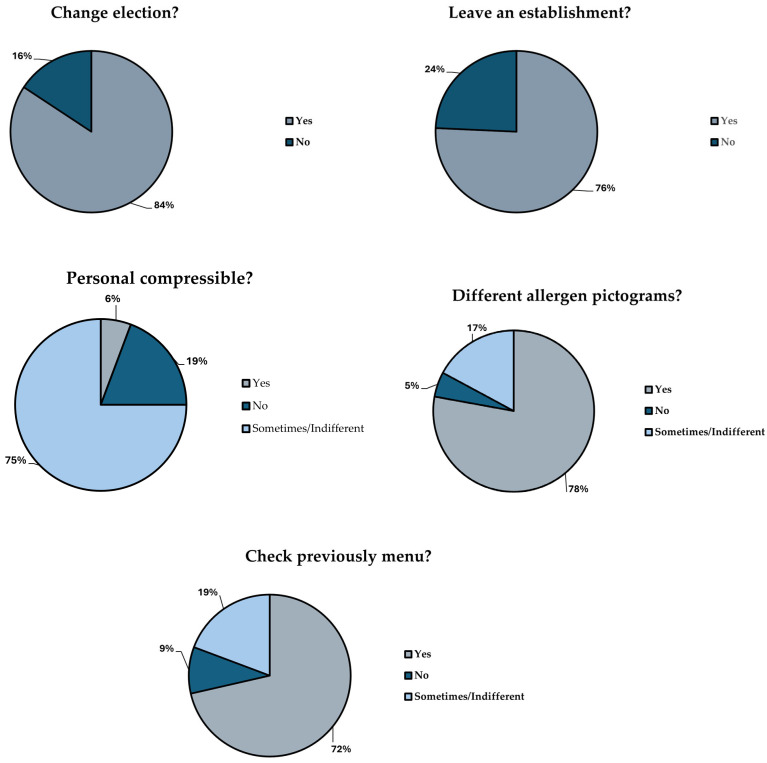
Answers to questions related to “Food safety outside the house” and “Collective catering and restaurants”.

Answers for the questions of sections related to “safety outside of the house and collective catering” are compiled on Figure 4. Overall, 85.8% of participants felt that there have been times when they felt excluded for their allergy problems, demonstrating a significant difference between genders, with more women feeling more excluded than men (*p* = 0.030). In total, 74.6% of participants had developed an allergic reaction when eating out and 84.3% had to change their food choice even when the allergen menu indicated it was safe to consume. In addition, 74.6% of participants have sometimes left a food establishment due to not being able to eat anything safe for them, with this being more frequent in those participants diagnosed in their childhood with respect to those diagnosed during their adolescence (*p* = 0.007).

According to the answers, only 5.2% of the staff of restaurants showed empathy or understanding with the allergy situation; however, most of them showed indifference in the matter (76.9%). The way that allergens are presented in each establishment is different, and this fact made 76.9% of the participants upset. It was frequent (72.4% of participants) for them to check the menu beforehand.

## 4. Discussion

### 4.1. Allergies and Life Quality

The results obtained in this study agree with the consequences in the quality of lives of people with food allergies previously reported by [17]. The answers showed how food allergies not only affected the physical level but also directly affected their social and emotional levels, which led to their life quality deteriorating.

Food allergies induce a persistent state of concern and vigilance, both due to their direct impact on health and the uncertainty of not always knowing the specific allergens involved, as well as the risk of experiencing increasingly severe reactions over time. The results of this exploratory study point out that depending on the age of diagnosis, the perception and fear of certain reactions changes. Those participants diagnosed as adults, in general, felt more fear of having an allergic reaction than those diagnosed during childhood or adolescence. This fact could be associated with them having dealt with food allergies for a longer period and having acquired certain habits of restrictions since they were young. Food allergies have a significant effect on activities of families of food allergic children [18,27,28].

The main factors that adversely affected the quality of life for individuals with alimentary allergies included the necessity to implement dietary restrictions (to prevent allergen exposure), the limited availability of treatment options, and the anxiety over unintentional contact with allergens [9,29,30,31,32].

The answers obtained from the questionnaire showed that in all variables asked, there was higher worry, discomfort, or even, on occasions, fear associated with everyday activities, which affected their social relationships as well. Living with allergies imposes restrictions on daily life, as any food-related activity can pose a risk. Many activities involve food forces, in many occasions, to turn down plans and social events, which can lead to a feeling of isolation or exclusion [9,17,33]. According to the results of the survey, women tend to feel more excluded than men. The results match with the significant differences found in the revision made by Rosser [29] who also pointed out that women tended to have more anxiety and fear of allergic reactions; however, the results could be altered depending on the kind of food that caused the allergy [28]. More studies focused on the effect of gender are required.

### 4.2. Food Labeling

The life quality of people with food allergies could improve if, when making laws, their opinion related to how the information available to consumers was taken into consideration. Furthermore, it can be used as a tool to avoid accidental exposure to allergens and a way to contribute to warranting food safety.

Prior to the publication of the EU Regulation 1169/2011, Cornelisse–Vermaat [34] conducted an intercultural study in different European countries (Greece and the Netherlands) in order to analyze the perception of food labeling in consumers with food allergies. Their results showed that in general, consumers with food allergies were dissatisfied with labeling practices. They found the information to be unclear or insufficient, leading to personal stress and feelings of insecurity.

People with food allergies and the people surrounding them would invest more time getting groceries as it implied reading all food labels, even if they consumed the product before, as there could be changes to their ingredient list [34]. Even with the application of Regulation 1169/2011, this seems to not have changed with time, as shown in the results of the survey, in which having to check labels continuously proves to be very upsetting for people with allergies as it requires spending more time than needed.

On many occasions, food labeling influences food choices since labels are incomplete or not clear enough and, as can be seen in the answers of the survey, this usually upsets individuals with food allergies. The perception of the lack of clarity in the way information is presented seems to be common in people with food allergies, even with the application of the current regulations. Individuals with food allergies consider that on many occasions, food labels are confusing or not clear enough to read, as allergens could be hidden in additives or thickening agents [33,34].

The main complaints pointed out in the paper by Cornelisse–Vermaat [34] referred to the format of the label, remarking that either the font used was too small or there were issues that were difficult to read (associated with the contrast of colors used in certain materials). Regulation 1169/2011 established in article 13 that the font size in labels and packages, as well as the way of presenting the mandatory information for consumers, should be included in a visible place that is easy to read and indelible. In any case, this information should be concealed, covered, or separated by any other indication or image or by any other intervening material. The way to highlight the existence of allergens was included in article 21. Thus, they must be highlighted by means of a typographic composition that clearly differentiates it from the rest of the ingredients, for example, by means of the typeface, style, or background color and in the case of not containing a list of ingredients, by means of the word “contains” followed by the name of the substance or product listed in Annex II of the aforementioned regulation [12].

The presence of explanatory symbols on labels, indicating whether an allergen is present in the product, was considered a positive tool for consumers [34]; this matches with the results of the present questionnaire, as they allow a better identification of the allergen. Article 9 of Regulation 1169/2011 introduces the possibility of using pictograms and symbols provided that the same level of information is ensured as with the use of letters and numbers [12]. However, the text does not indicate the form or format of presentation. To avoid ambiguities and as a complement to the list of ingredients, it would be advisable to homogenize the pictograms and symbols used since they are many and diverse.

On the other hand, the inclusion of terms such as ’May contain traces of...’ or similar on labels annoys people with food allergies and limits their consumption of certain foods. The appearance of this warning is necessary under Regulation (EC) No 852/2004 on the hygiene of foodstuffs [11] and Regulation (EC) No 178/2002, which lays down the general principles and requirements of food law [35], establishes the European Food Safety Authority (EFSA), and sets procedures in matters of food safety to ensure the safety of marketed products whenever the producer cannot guarantee the absence of the allergen in the food.

Therefore, precautionary allergen labeling (PAL) aims to warn consumers of the possible unintentional and unavoidable presence of an allergen, for example, due to potential cross-contamination. However, there are operators in the market who highlight the allergens listed in the precautionary allergen labeling and others who do not, so a uniform criterion at the national level is required [36].

At the community level, there is also no regulation governing PAL. However, its development is planned to ensure that this information does not mislead consumers and is neither ambiguous nor confusing, as indicated in Article 36.3.a of Regulation 1169/2011 [11]. This new regulation will be based on relevant scientific data. The large amount of data and new technologies can be applied to assess the risk of the presence of unwanted allergens in food and thus determine quantitative reference parameters (reference doses) or reconsider existing ones, which would represent an improvement [37].

It may also be an advancement to standardize the presentation of PAL, with the possibility of globally implementing the guidelines established by the Codex Alimentarius Commission [38]. Among these guidelines, the inclusion of the phrase ’May contain’ (or equivalent words) followed by the identified allergens is highlighted. The use of these terms by the economic operator solely to avoid unnecessary risks can limit food selection options for allergic individuals. The overuse of PAL can be reduced if its use is restricted to situations where the unintentional presence of an allergen cannot be controlled and is always based on a risk assessment that includes the reference doses established by national authorities. The establishment of education and information programs on PAL is essential to ensure its understanding by consumers and its use by food business operators [38].

### 4.3. Collective Catering and Food Allergies

Eating out means assuming a very important risk, being constantly alert, not always being able to try the dishes served, and, on many occasions, having to check the food they are going to eat. This, as the results of the questionnaire have shown, is very upsetting for the participants and, according to different studies, can generate anxiety, stress, and fear, which leads many of these people not to eat out or to reject plans in which food is involved [17,33].

In addition to the risk involved in eating out, people with food allergies may feel that not enough importance is given to their problem. In this study, people who are diagnosed in adolescence are extremely upset by this and it may be because this stage coincides with the time when they become more independent, gain autonomy in decision making, and have a social life without parental supervision [39]. The fact that their allergy problem is not taken seriously can severely limit their social life and influence the independence they are beginning to have [39].

There is a general concern around the correct communication of food allergies. Those diagnosed in childhood tend to be much more afraid than the other groups to eat out despite explaining their food restrictions in advance. They are also more afraid of eating something by accident and are the group that most leave a place because it does not accommodate their allergies. This may be because during their childhood, when they ate outside, it was their parents or relatives who were responsible for explaining and managing their allergy problem, avoiding those situations that could endanger the allergic person [33,40], which would make them feel safer.

The results of the questionnaire show that people with food allergies do not feel safe eating out and that the majority have suffered an allergic reaction when eating out. The lack of training of food handlers in food safety, more specifically in cross contamination, and the difficulties they experience may explain the lack of confidence in eating out, as indicated by several studies [33,39,41]. The perception that people with a food allergy have about food safety is often feelings of insecurity [40]. Consumers believe that foodservice personnel have limited knowledge of food intolerances and allergies. These staff usually have basic training in food hygiene, but the length or extent of training on allergens and special dietary requirements depends on the training organization itself. Even those who have done some training mentioned that they did not feel sufficiently prepared to ensure that serving a customer with an allergy is safe.

Regulation (EC) No 852/2004 on the hygiene of foodstuffs [10], in its Annex II, Chapter XII, makes it mandatory for food handlers to be trained according to their job activities and to comply with all national legislation requirements regarding training programs for workers in certain food sectors. However, there is no specific European or national standard regulating the training requirements such as content, exams, instructors, and the method of delivering the training. All of this depends on the training company, which also does not need any official approval or authorization to provide training to the staff of food establishments [42].

This results in a lack of standardization in food hygiene training in general and allergen training in particular. Since the duration or extent of allergen training is not legally specified, it may be insufficient. This could explain why those handlers who had received training in this area did not feel adequately prepared to ensure the safety of the food served to customers. Therefore, to unify and improve the training of handlers, the content, duration of training courses, evaluation, instructors, and method of delivery should be legally and specifically regulated. Improving training would positively impact food safety and increase the confidence of both the handlers and the consumers.

It has been documented that people with food allergies prefer to have written information when choosing food to avoid discussing their problem with the staff serving them [40,43] either for fear of being considered problematic customers [44,45] or because they do not feel understood in the situation, as reflected in the results of this survey. Therefore, to facilitate the understanding of the information, the use of pictograms helps in the choice of food outside the home.

The results of this study indicate that the way in which allergens are displayed matters because it is usually the first thing that an allergic person notices and what makes him or her decide on one place or another, especially for those diagnosed as adults, although also for those diagnosed in childhood. However, most respondents are annoyed by the way allergens are displayed, being different depending on each establishment. RD 126/2015 requires allergens to be declared, among others, in foods presented unpackaged for sale to the final consumer and to mass caterers, although it does not indicate how to do it, so each restaurant has a different criterion [15].

Despite the above, people with food allergies usually ask and confirm that the preparation of the dish is suitable for them, employing verbal communication with the person who serves them, which is essential to ensure safety since the food is prepared in a different way than the one prepared at home [40,44]. This sometimes leads, as shown by the answers of the questionnaire, to them changing their food choice because although the allergens on the menu indicated that it was safe, the staff may have indicated otherwise.

The answers obtained from this questionnaire support the previous idea proposed by Figueroa [40] that it is necessary to improve communication between staff and consumers, as well as training the staff.

Recent publications [41,46,47,48] in the area show how this concern and measures are beginning to be implemented in mass catering. In addition, more information should be provided to customers on menus about the presence of allergens [48] and food hygiene training should be homogenized by reinforcing those sections related to food allergens. This would contribute to more rigorous and accessible training, as demanded even by the sector itself [49].

## 5. Conclusions

Based on the data derived from this exploratory study, it can be concluded that individuals with food allergies generally perceive food safety with a sense of insecurity, particularly when eating outside the home. The need to constantly check all purchased and consumed foods, as well as their labels, has a negative impact on emotional health and social relationships, in addition to posing a risk to physical health.

Differences have been observed in the impact on quality of life depending on gender and age of diagnosis, as well as in the perception of various factors evaluated by the questionnaire. Men reported greater fear of accidentally consuming something that could trigger an allergic reaction, whereas women felt more socially excluded due to their allergies.

Those diagnosed in childhood experienced greater fear of accidental exposure and were more likely to leave a situation that did not accommodate their allergies. Adolescents diagnosed with food allergies were the most affected by the underestimation of their condition. Adults diagnosed later in life exhibited the highest levels of fear regarding allergic reactions

Regarding food labeling, although regulatory progress has been made, survey results highlight the need for a more standardized and strictly monitored framework to ensure the safety of individuals with various food allergies, facilitating allergen-free consumption. This includes revising how allergen information is presented to consumers and incorporating pictograms, which are regarded as useful and effective tools.

In the food service sector, it is essential to improve communication between food service staff and consumers, as well as to train restaurant personnel and raise awareness of their critical role in ensuring food safety for individuals with food allergies. Enhancing this aspect can transform dining out into a safer and more satisfying experience, thereby improving quality of life and social interactions.

This study was conducted using the shortened FAQLQ questionnaire targeted at adults. However, other versions of both shortened and full-length questionnaires exist to assess the quality of life of different population groups. Conducting these additional surveys would provide a more comprehensive understanding of the quality of life of individuals with food allergies in Spain and other regions, also enabling greater generalizability of the results. Furthermore, additional research is needed to assess the level of allergen-related knowledge among food service personnel.

## Figures and Tables

**Table 1:** Items in food allergy quality of life questionnaire.

FAQLQ * nº	
	How troublesome do you find it, because of your food allergy, that you …
FAQLQ 01	Must always be alert as to what you are eating?
FAQLQ 09	Are less able to taste or try various products when eating out?
FAQLQ 11	Must personally check whether you can eat something when eating out?
FAQLQ 13	That the ingredients of a product change?
	How troublesome is it, because of your food allergy …
FAQLQ 14	That labels are incomplete?
FAQLQ 18	That people underestimate your problems caused by food allergy?
FAQLQ 19	that it is unclear to which foods you are allergic?
	How worried are you because of your food allergy …
FAQLQ 22	About your health?
FAQLQ 23	Thar the allergic reactions to foods Will become increasing severe?
	How frightened are you because of your food allergy …
FAQLQ 24	Of an allergic reaction?
FAQLQ 25	Of accidentally eating the wrong food?
FAQLQ 26	Of an allergic reaction when eating out despite the fact that your dietary restrictions have been discussed beforehand?

* Social and dietary limitations (ítems 01, 09, and 11); risk (ítems 13, 14, and 18); health (ítems 19, 22, and 23); and emotional impact (24–26). Source adapted from Coelho [18].

**Table 2:** Schematic design of the type of questions and answers in the FAQLQ questionnaire.

The Following Questions Concern the Influence Your Food Allergy Has on Your Quality of Life. Answer Every Question by Marking the Appropriate Box. You May Choose from One of the Following Answers							
	Not	Barely	Slightly	Moderately	Quite	Very	Extremely
FAQLQ (nº)							

**Table 3:** Questions related to food safety, labeling, and training of collective catering professionals.

	Item
Labeling	How troublesome do you find it, because of your food allergy, that labels present: “May contain traces of…”?
	How useful do you find pictograms on food labels? (e.g., gluten-free/lactose-free…)
	Where do you feel that your allergies have not been given importance?
Food safety	Do you feel safe eating out if you don’t prepare your own food?
outside home	Have you ever felt left out because of your allergy problems?
	Have you had any allergic reactions while eating outside home?
	Have you ever had to change your food choices even though the allergen menu said it was safe?
Collective catering	Have you ever had to leave a place because it didn’t accommodate your allergies?
Restaurants	When you eat out, do you feel that restaurant staff are considerate or understanding of your allergies?
	How troublesome do you find it, because of your food allergy, that allergens are presented differently in each establishment?
	When you go to eat at a new restaurant, do you look at the menu and the allergen menu before you go?

**Table 4:** Perfil de los participantes en el cuestionario (*n =* 134).

Item	Category	% Answer
Gender	Women	73.1
	Men	26.9
Age interval	18–24	35.1
	25–34	20.1
	35–44	19.4
	45–54	20.1
	55–64	4.5
	>65	0.7
Age of allergy diagnosis	Childhood	56.7
	Adolescence	12.7
	Adult	12.7

**Table 5:** *p* values affect gender and age of diagnosis in the variables of the questionnaire.

Variable Category	Item	*p* Value Gender	*p* Value Age of Diagnosis
	Gender	-	<0.001
	Age of diagnosis	<0.001	-
	Healthy and balanced diet	0.274	0.601
Nutrition	Nutritional deficiency	0.091	0.720
	Easy food replacement	0.652	0.721
	FAQLQ 1	0.680	0.315
Social and dietary	FAQLQ 9	0.910	0.433
limitations	FAQLQ 11	0.943	0.137
	FAQLQ 13	0.954	0.212
Risk	FAQLQ 14	0.538	0.568
	FAQLQ 18	0.504	0.052
	FAQLQ 19	0.697	0.366
Health	FAQLQ 22	0.888	0.717
	FAQLQ 23	0.136	0.129
	FAQLQ 24	0.492	0.048
Emotional impact	FAQLQ 25	0.003	0.005
	FAQLQ 26	0.082	0.124
Labeling	Label “traces of…”	0.865	0.607
	Food label pictograms	0.095	0.238
	Safe eating outside	0.969	0.086
Food safety outside home	Feeling left out because of allergies	0.030	0.389
	Allergic reaction eating away from home	0.698	0.179
	Change election	0.731	0.634
Collective catering	Leave an establishment	0.339	0.007
Restaurants	Personal compressible	0.237	0.051
	Different allergen pictograms	0.155	0.321
	Check previously menu	0.278	0.112

**Table 6:** Percentage of answers in the shorter version of FAQLQ for adults *(n* = 134).

Question/%	Not	Barely	Slightly	Moderately	Quite	Very	Extremely
FAQLQ 1 Be alert eating	7.5	5.2	7.5	11.2	17.9	20.9	29.9
FAQLQ 9 Be less able to try products	2.2	2.2	6.7	9.7	11.9	20.1	47
FAQLQ 11 Check whether you can eat	4.5	1.5	3	6.7	13.4	16.4	54.5
FAQLQ 13 Ingredients of a product change	0	0	1.5	0.7	10.4	12.7	74.6
FAQLQ 14 Labels incomplete	1.5	0	0	3.7	13.4	20.9	60.4
FAQLQ 18 Underestimate your allergy	8.2	2.2	7.5	9	17.9	21.6	33.6
FAQLQ 19 Unclear which foods you are allergic?	7.5	5.2	7.5	11.2	17.9	20.9	29.9
FAQLQ 22 Worried about your health?	0.7	3	5.2	7.5	23.9	23.1	36.6
FAQLQ 23 Allergic reactions became severe	1.5	0.7	2.2	5.2	14.9	20.1	55.2
FAQLQ 24 Frightened allergic reaction	0.7	1.5	0.7	8.2	12.7	20.9	55.2
FAQLQ 25 Frightened accidentally eating wrong food	1.5	0.7	1.5	4.5	14.9	23.1	53.7
FAQLQ 26 Frightened allergic reaction eating out	0.7	0.7	0.7	3.7	7.5	17.9	68.7

**Table 7:** Effect of gender in question FAQLQ 25—How frightened are you because of your food allergy: Of accidentally eating the wrong food (% answers).

FAQLQ 25/(% Answers)	Men	Women	*n*	*p* Value
Not	0	2	2	
Barely	0	1	1	
Slightly	2.8	1	2	
Moderately	2.8	5.1	6	0.003
Quite	2.8	19.4	20	
Very	16.7	25.5	31	
Extremely	75	45.9	72	

**Table 8:** Effect of the age of diagnosis on questions FAQLQ 24 and 25. (% answers).

Question	Childhood	Adolescence	Adulthood	*n*	*p* Value
FAQLQ 24					
Not	0	0	2.4	4	
Barely	1.3	0	2.4	2	
Slightly	1.3	0	0	1	
Moderately	6.6	0	14.6	11	0.048
Quite	13.2	5.9	14.6	17	
Very	22.4	17.6	19.5	28	
Extremely	55.3 ^ab^	76.5 ^b^	46.3 ^a^	74	
FAQLQ 25					
Not	1.3	0	2.4	2	
Barely	0	0	2.4	1	
Slightly	2.6	0	0	2	
Moderately	1.3 ^a^	5.9 ^ab^	9.8 ^b^	6	0.005
Quite	7.9 ^a^	0 ^a^	34.1 ^b^	20	
Very	26.3 ^ab^	35.3 ^b^	12.2 ^a^	31	
Extremely	60.5 ^b^	58.8 ^ab^	39.0 ^a^	72	

^a,b^ Different superscripts represent significant differences among columns (*p* ≤ 0.052).

**Table 9:** Answers related to labeling and food safety.

Question	Not	Barely	Slightly	Moderately	Quite	Very	Extremely
Label “traces of…”	6	0.5	3	5.2	18.7	21.6	44.8
Food label pictograms	7.5	3	5.2	9.7	23.1	24.6	26.9
Safe eating outside	22.4	17.9	12.7	25.4	14.2	4.5	3

**Table 10:** Means of answers related to FAQLQ, labeling and food safety.

Question	Mean	SD ^1^	SEM ^2^
FAQLQ 1 Be alert eating	5.28	1.629	0.141
FAQLQ 9 Be less able to try products	5.09	1.870	0.162
FAQLQ 11 Check whether you can eat	5.75	1.568	0.135
FAQLQ 13 Ingredients of a product change	5.90	1.608	0.139
FAQLQ 14 Labels incomplete	6.58	0.825	0.071
FAQLQ 18 Underestimate your allergy	6.32	1.080	0.093
FAQLQ 19 Unclear which foods you are allergic?	5.25	1.850	0.160
FAQLQ 22 Worried about your health?	5.66	1.392	0.120
FAQLQ 23 Allergic reactions became severe	6.13	1.265	0.109
FAQLQ 24 Frightened allergic reaction	6.14	1.221	0.105
FAQLQ 25 Frightened accidentally eating wrong food	6.15	1.217	0.105
FAQLQ 26 Frightened allergic reaction eating out	6.45	1.045	0.090
Label “traces of…”	5.74	1.636	0.141
Food label pictograms	5.19	1.754	0.151
Safe eating outside	3.16	1.664	0.144

^1^ SD: Standard deviation; ^2^ SEM: Standard error of mean.

## Data Availability

The data presented in this study are available on request from the corresponding author.
